# Purification of the Recombinant Green Fluorescent Protein Using Aqueous Two-Phase System Composed of Recyclable CO_2_-Based Alkyl Carbamate Ionic Liquid

**DOI:** 10.3389/fchem.2018.00529

**Published:** 2018-10-31

**Authors:** Cher Pin Song, Poh En Liew, Zora Teh, Schian Pei Lim, Pau Loke Show, Chien Wei Ooi

**Affiliations:** ^1^Chemical Engineering Discipline, School of Engineering, Selangor, Malaysia; ^2^Advanced Engineering Platform, School of Engineering, Monash University Malaysia, Selangor, Malaysia; ^3^Bioseparation Research Group, Department of Chemical and Environmental Engineering, Faculty of Engineering, University of Nottingham Malaysia, Selangor, Malaysia

**Keywords:** purification, green fluorescent protein, aqueous two-phase system, dialkyl carbamate, poly(propylene glycol), recycling

## Abstract

The formation of aqueous two-phase system (ATPS) with the environmentally friendly and recyclable ionic liquid has been gaining popularity in the field of protein separation. In this study, the ATPSs comprising *N*,*N*-dimethylammonium *N*′*,N*′-dimethylcarbamate (DIMCARB) and thermo-responsive poly(propylene) glycol (PPG) were applied for the recovery of recombinant green fluorescent protein (GFP) derived from *Escherichia coli*. The partition behavior of GFP in the PPG + DIMCARB + water system was investigated systematically by varying the molecular weight of PPG and the total composition of ATPS. Overall, GFP was found to be preferentially partitioned to the hydrophilic DIMCARB-rich phase. An ATPS composed of 42% (w/w) PPG 1000 and 4.4% (w/w) DIMCARB gave the optimum performance in terms of GFP selectivity (1,237) and yield (98.8%). The optimal system was also successfully scaled up by 50 times without compromising the purification performance. The bottom phase containing GFP was subjected to rotary evaporation of DIMCARB. The stability of GFP was not affected by the distillation of DIMCARB, and the DIMCARB was successfully recycled in three successive rounds of GFP purification. The potential of PPG + DIMCARB + water system as a sustainable protein purification tool is promising.

## Introduction

Green fluorescent protein (GFP) has been widely applied in the cellular and molecular biology research due to its unique properties such as the intense fluorescence visibility, high thermal stability, and the adjustable fluorescence intensity via a proper manipulation of the protein structure (Skosyrev et al., 2003; Li et al., 2009; Quental et al., 2015). Additionally, GFP can be easily quantified via spectrofluorimetric assay, making it a good candidate as a biosensor (Wouters et al., 2001) and biomarker (Gerisch et al., 1995) in the biotechnological application. The recombinant GFP has been successfully expressed by various organisms, including *Escherichia coli* (*E. coli*) (Lo et al., 2018), zebrafish (Amsterdam et al., 1996), *Drosophila* (Wang and Hazelrigg, 1994), and yeast (Amsterdam et al., 1996). Nevertheless, the purification of GFP with the conventional chromatographic techniques generally involves complex and tedious operations, resulting in a higher purification cost (Deschamps et al., 1995; Cabanne et al., 2005; McRae et al., 2005; Zhuang et al., 2008).

Aqueous two-phase system (ATPS) has been widely viewed as a potential alternative method for the separation of biomolecules. The advantages of ATPS include the high extraction efficiency, the cost effectiveness, and the simplicity of operation. This type of liquid-liquid extraction is commonly exploited for the primary recovery and purification of valuable biological products such as proteins (Merchuk et al., 1998), enzymes (Kroner et al., 1982), nucleic acids (Gomes et al., 2009), and viruses (Liu et al., 1998). ATPS is conventionally made of two types of incompatible polymers, or a polymer coupled with a salting-out inducing salt; the concentrations of phase-forming components in an aqueous solution must exceed the threshold value. ATPS has been widely perceived as a biocompatible medium for preserving the biological properties of biomolecules, owing to the large proportion of water content in both phases (Yao et al., 2018). The extraction of GFP has been successful achieved using the traditional ATPSs consisting of phase-forming components such as polymer, surfactant, alcohol, and inorganic salts (Jain et al., 2004; Johansson et al., 2008; Li and Beitle Robert, 2008; Samarkina et al., 2009; Lopes et al., 2011; Lo et al., 2018). However, the limited polarity range of the coexisting phases and the poor recyclability of the conventional phase-forming components have constituted a major bottleneck that hampers the vast use of these conventional ATPSs (Hatti-Kaul, 2000).

Over the past decade, ionic liquid (IL) has been envisaged as an alternative phase-forming component of ATPS due to its highly tunable properties (Freire et al., 2012). IL is a type of molten organic salt with a melting point below 100°C. By properly selecting the cation and anion counterparts, the resultant ILs possess the desired polarity and affinity suitable for the separation of protein in ATPS. In comparison to the conventional polymer-based ATPS, the flexibility of IL-based ATPS allows a better design of the separation system for the target protein in a highly complex crude mixture. Nonetheless, the wide implementation of IL in liquid-liquid extraction is still restricted by the synthesis cost of IL, which is generally more expensive than the conventional phase-forming components (Plechkova and Seddon, 2008). Moreover, some of the conventionally used ILs (i.e., imidazolium- and pyridinium-based ILs) are reported to be highly toxic (Docherty and Kulpa, 2005). With the rising environmental consciousness in public, the application of environmentally benign ILs (e.g., cholinium- and amino acids-based ILs) in forming ATPS has been on the rise (Song et al., 2015, 2017, 2018a).

The CO_2_-based alkyl carbamate IL, which is formed by the combination of CO_2_ with dimethylamine (Bhatt et al., 2006; Chowdhury et al., 2010; Idris et al., 2014; Vijayaraghavan and MacFarlane, 2014), has recently emerged as a potential phase-forming component of IL-based ATPS. In general, the synthesis of the alkyl carbamate IL is considerably simpler and cheaper than the conventional ILs (Kreher et al., 2004). It has been reported that the alkyl carbamate IL possesses the characteristics of biodegradability and biocompatibility (Stark et al., 2009). More importantly, the CO_2_-based alkyl carbamate IL can be distilled at a relatively low temperature under vacuum condition, thereby allowing a simple recovery of IL for the subsequent extraction process (Vijayaraghavan and MacFarlane, 2014). Recently, our group reported a novel type of IL + polymer ATPS comprising *N*,*N*-dimethylammonium *N*′*,N*′-dimethylcarbamate (DIMCARB, i.e., the simplest form of CO_2_-based alkyl carbamate IL) and the thermo-responsive poly(propylene) glycol (PPG) (Song et al., 2018b). Both DIMCARB and PPG used in this ATPS can be recovered via rotary evaporation and thermo-separation, respectively, for a viable recycling of phase-forming component.

Here, the application of DIMCARB + PPG + water systems for separating the target biomolecule from a real crude protein mixture was reported for the first time. The purification of recombinant GFP from the clarified lysate of microbial feedstock was performed using ATPSs made of DIMCARB and PPG. The stability and partition behavior of GFP in the ATPSs were studied, and the composition of ATPS was optimized for the purification of GFP. To evaluate the sustainability of this ATPS for practical use, DIMCARB was also recycled for several rounds of GFP purification.

## Materials and methods

### Materials

DIMCARB, and PPG (molecular weight of 400, 700, and 1,000 g.mol^−1^) were obtained from Sigma-Aldrich (St. Louis, U.S.A.). Tris base (ULTROL grade) was obtained from CalBiochem (San Diego, U.S.A.). Luria-Bertani (LB) broth, kanamycin sulfate, chloramphenicol, ethanol, isopropyl β-D-1-thiogalactopyranoside (IPTG), methanol, and acetic acid were purchased from Merck (Darmstadt, Germany). Coomassie Brilliant Blue R-250 (CBB-R250) staining solution were sourced from Bio-Rad Laboratories (Singapore). Protein marker (ExcelBand™ 3-color Broad Range) with the molecular weight ranging from 5–245 kDa was acquired from SMOBiO Technology (Taiwan). All chemicals were of analytical reagent grade.

### Methods

#### Production of recombinant GFP

The GFP was expressed by *E. coli* strain BL21(DE3)pLysS transformed with pET28a-GFP plasmid. The cells were cultured at 30°C in LB broth medium containing 50 μg/ml kanamycin and 50 μg/ml chloramphenicol. When the optical density (OD_600_) of cell culture reached 0.7–0.9, 0.5 mM IPTG was added to the culture for the induction of GFP expression. The cell culture was incubated in an orbital shaker for another 12 h at 30°C and 200 rpm. Then, the culture broth was centrifuged at 4,000 rpm and 4°C for 20 min. The harvested cell pellets were resuspended in 50 mM Tris-HCl (pH 8) buffer, and the concentration of biomass was adjusted to 10% (w/v). The disruption of cells was performed using an ultrasonic homogenizer (Cole-Palmer, U.S.A.) equipped with a horn-tip of 3 mm diameter (Model KH-04710-42, Cole-Parmer, U.S.A.) and operated at a frequency of 20 kHz, 40% amplitude for 40 min in pulse mode (Lo et al., 2016). Finally, the ultrasonicated solution was centrifuged for 10 min at 14,000 rpm and 4°C. The supernatant containing the soluble GFP was collected and used as the feedstock for ATPS.

#### Protein quantification

The concentration of GFP was determined spectrofluorometrically using a standard curve of pure GFP. The preparation of the pure GFP is described elsewhere (Lo et al., 2018). In brief, 100 μl of the sample was first loaded in a black microtiter plate. The relative fluorescence unit (RFU) of the sample were measured using a spectrofluorometer (Infinite® 200 PRO, Tecan) at the excitation wavelength of 448 nm and the emission wavelength of 512 nm. The concentration of protein in the sample solution was estimated from the polyacrylamide gel using densitometric method as described (Lee et al., 2015). In the gel, the protein bands in a sample lane were evaluated individually based on the intensity ratio of a single band to the total bands in a lane. The intensity of control band (i.e., pure GFP) was used in the calculation of the protein concentration.

#### Sodium dodecyl sulfate polyacrylamide gel electrophoresis (SDS-PAGE)

Prior to the SDS-PAGE analysis, the protein samples were subjected to ethanol precipitation for the removal of phase-forming components (e.g., ionic DIMCARB) that could interfere the electrophoresis. A mixture containing one part of sample solution and nine parts of chilled absolute ethanol was first prepared. The mixture was vortexed vigorously, before being stored at −20°C for 60 min. Next, the solution was centrifuged at 15,000 rpm for 10 min. After discarding the supernatant, the pellet was resuspended in the chilled ethanol and the solution was centrifuged again. The washing step was performed twice, and the pellet was then dried at room temperature for 10 min. Lastly, the pellet was re-solubilized in Tris-HCl buffer (pH 8; 50 mM).

The SDS-PAGE was conducted using a 12% (w/v) resolving gel in combination with a 5% (w/v) stacking gel (Laemmli, 1970). The thickness of the polyacrylamide gel was 1 mm. The electrophoresis was conducted at 180 V for 60 min using an electrophoresis unit (Mini Protean™ 3, Bio-Rad, U.S.A.). After the electrophoresis, the gel was stained with Coomassie Brilliant Blue R-250 for 45 min. Subsequently, the gel was destained with a destaining solution made of 10% (v/v) methanol and 10% (v/v) acetic acid until a clear background in the gel was formed. The protein bands on the gel were visualized and analyzed using a gel imaging system (Gel Doc™ XR +, Bio-Rad).

#### Partitioning of GFP in ATPSs

ATPS was prepared in a 2-ml micro-centrifuge tube by adding the appropriate amounts of PPG, DIMCARB, buffer solution (50 mM Tris-HCl) and 10% (w/w) crude feedstock, with the pH of final mixture was adjusted to the optimum pH. Next, the system was mixed thoroughly using a vortex mixer prior to settling for 3 h to attain the phase equilibrium. The temperature of the system was maintained at 25°C during the incubation in a thermostatic bath. Subsequently, the system was centrifuged at 4,000 rpm for 10 min to achieve a complete phase separation. The partition coefficient of GFP (*K*_GFP_) or total protein (*K*_protein_) was determined using Equation (1):

$$\begin{array}{r} K=\frac{C_{\text{B}}}{C_{\text{T}}} \end{array}$$

where *C*_B_ and *C*_T_ are the concentrations of proteins in the bottom and top phases, respectively.

Selectivity (*S*) was defined as the ratio of *K*_GFP_ to *K*_protein_, as shown in Equation (2):

$$\begin{array}{r} S=\frac{K_{\text{GFP}}}{K_{\text{protein}}} \end{array}$$

The yield of GFP partitioned to a specific phase of the system (*Y*) was calculated using Equation (3):

$$\begin{array}{r} Y\;\left(\%\right)=\frac{C_{\text{P}\left(\text{GFP}\right)}}{C_{\text{C}\left(\text{GFP}\right)}}\times 100 \end{array}$$

where *C*_P(GFP)_ and *C*_C(GFP)_ represent the concentration of GFP in the top phase or the bottom phase of ATPS and the crude feedstock, respectively.

## Results and discussion

### Stability test for GFP

**Figure 1:**
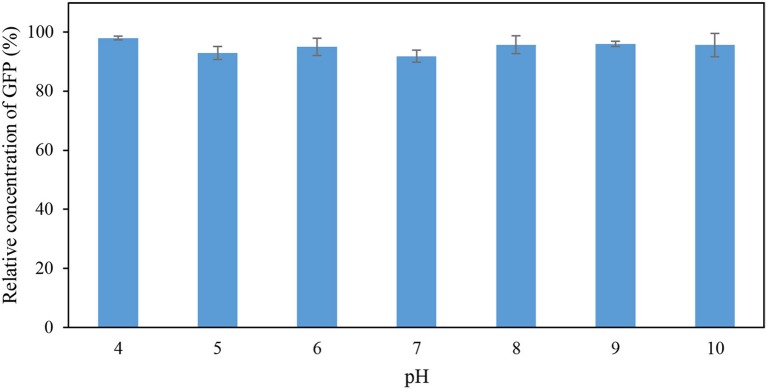
Percentage relative concentration of GFP incubated in 50 mM Tris-HCl buffer at different pH for 60 min at 25°C.

**Figure 2:**
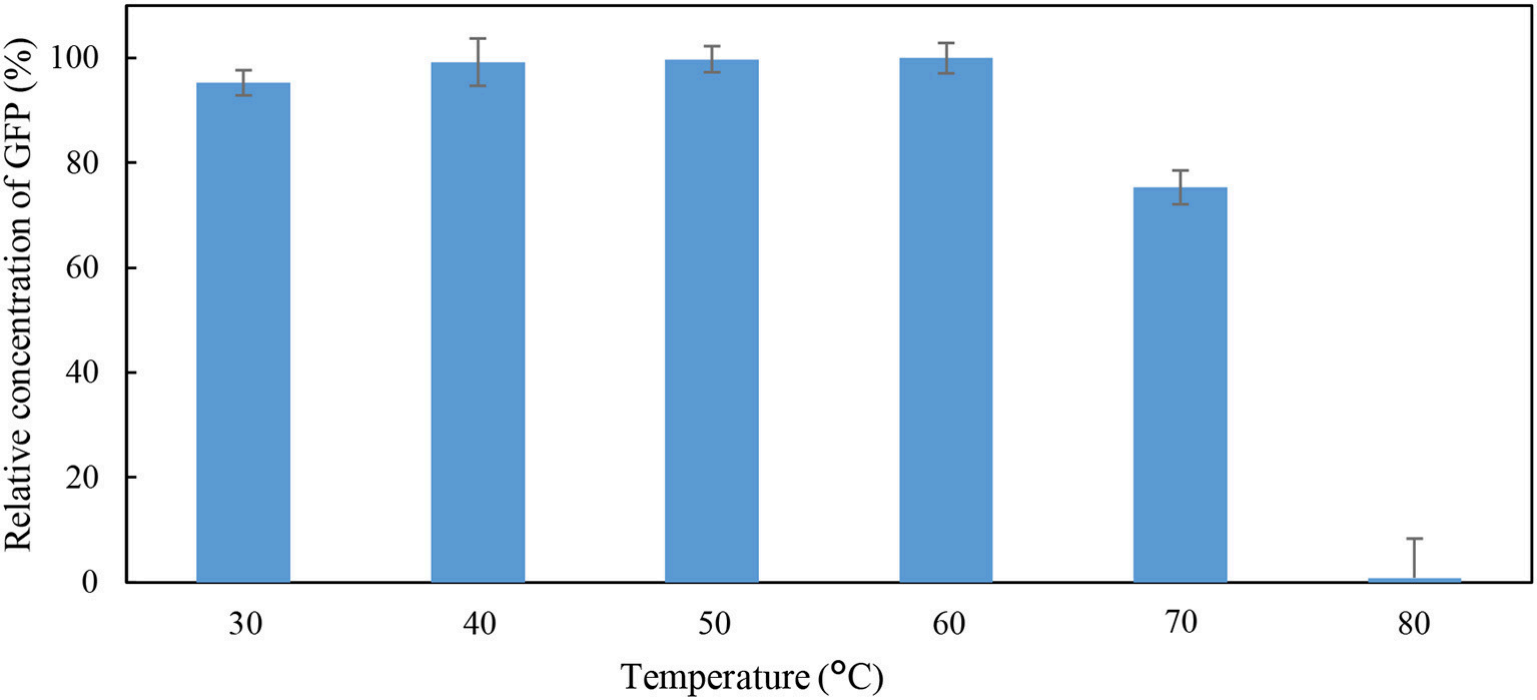
Percentage relative concentration of GFP incubated in 50 mM Tris-HCl buffer at pH 8 for 60 min at different temperature.

The effects of pH and temperature on the stability of GFP were evaluated by incubating the feedstock solution of GFP under different conditions of pH and temperature for 60 min. The GFP suspended in Tris-HCl buffer (pH 8; 50 mM) at 25°C for 60 min was used as the control. The stability of GFP was assessed using the indicator “percentage relative concentration of GFP,” which is calculated as the RFU of sample as a percent of the RFU of the control. The results are shown in Figures 1, 2. At pH ranging from 4 to 10, the percentage relative concentration of GFP was >91.8%, showing that the stability of the protein was well preserved. A previous study (Johansson et al., 2008) reported that GFP is stable in a broad range of pH (5.0–11.5). An extreme pH condition affects the structural stability and solubility of protein, leading to an irreversible denaturation of protein.

As shown in Figure 2, a low percentage relative concentration of GFP (i.e., 24.7%) was observed when the incubation temperature was raised to 70°C. Thus, it can be concluded that the GFP was stable when incubated at temperature below 70°C. The GFP was mostly denatured when the incubation temperature increased to 80°C, as indicated by the 0.89% of the relative concentration of GFP. Generally, the matured GFP is relatively stable and is able to fluoresce at temperature up 65°C (Tsien, 1998). An increase in incubation temperature promotes the unfolding of native secondary and tertiary structures of GFP (Penna et al., 2005). Bokman and Ward (1981) reported that the native secondary structure of GFP is essential to maintain the fluorescent form of the chromophore.

### GFP partitioning in PPG + DIMCARB + water system

The partitioning of proteins in ATPSs is typically governed by the interaction between phase-forming components and biomolecules (Tubio et al., 2004). In an ATPS, a protein will interact with the surrounding molecules through interactions such as hydrophobic interactions, hydrogen bonding, electrostatic interactions, steric effects, and van der Waals forces (Dreyer et al., 2009). To design the ATPS for an efficient separation of protein, it is important to understand the factors governing the partitioning of protein between phases in an ATPS.

**Figure 3:**
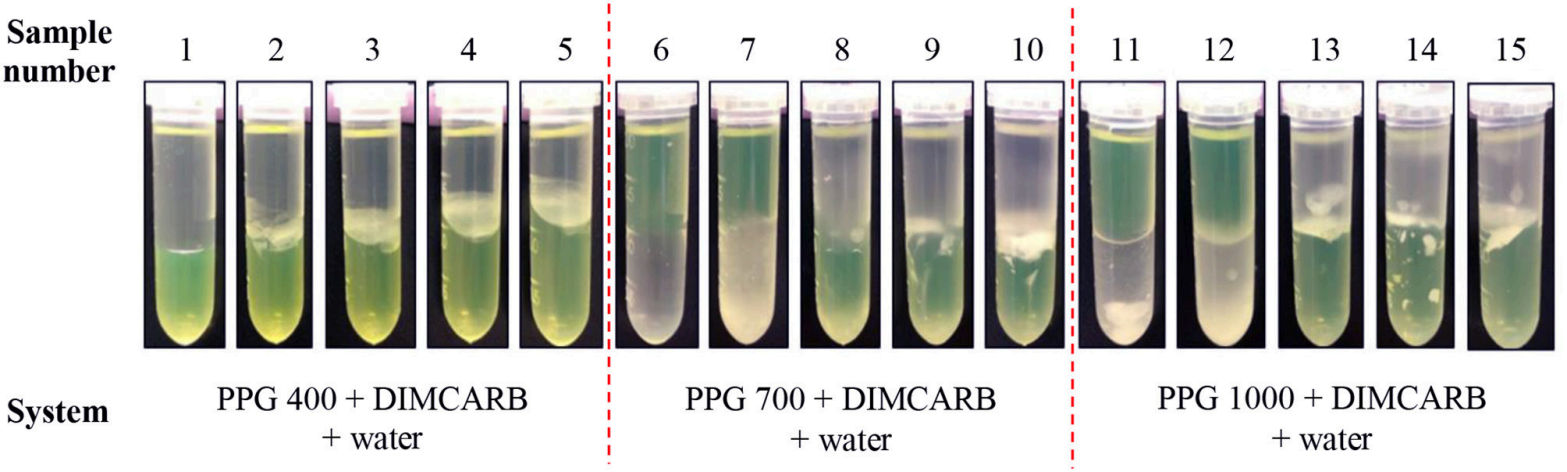
Photos of PPG 400/700/1000 + DIMCARB + water systems showing the partitioning of GFP between phases.

The partitioning of GFP in PPG + DIMCARB + water systems was investigated, and the results are presented in Table 1 and Figure 3. The compositions of the phase-forming components were selected based on the corresponding phase diagrams reported from our previous work (Song et al., 2018b). The concentrations of phase-forming components were varied according to the tie-line length (TLL). To exclude the effect of volume ratio on the GFP partitioning, the volume ratio of top and bottom phases was fixed at 1:1. From Table 1, majority of the investigated systems showed a positive value of log *K*_GFP_, indicating that the GFP was preferably partitioned to the DIMCARB-rich bottom phase. Among the investigated ATPSs, PPG 1000 + DIMCARB + water system at TLL = 97.4% (sample number 15) gave the highest *S* value (1237) and *Y*% of GFP in bottom phase (98.8%).

**Table 1:** Partition of GFP in PPG 400/700/1000 + DIMCARB + water systems at different phase compositions.

**System**	**Sampl number**	**TLL^a^**	**log *K*^b^**		***S***	***Y*** **(%)**	
			**GFP**	**Protein**		**Top phase**	**Bottom phase**
PPG 400 + DIMCARB + water	1	46.4	2.81	0.360	279.0	0.16	96.2
	2	69.8	2.78	0.219	365.8	0.15	89.2
	3	78.7	2.85	0.380	293.5	0.13	91.5
	4	85.6	2.83	0.365	294.7	0.13	90.9
	5	93.1	2.76	1.130	42.28	0.17	96.2
PPG 700 + DIMCARB + water	6	90.0	−2.88	−1.249	0.023	74.2	0.10
	7	92.9	−1.35	−0.858	0.325	76.6	3.45
	8	94.3	3.09	1.008	119.9	0.08	98.2
	9	95.2	3.09	0.974	131.1	0.08	95.3
	10	95.8	3.12	0.934	154.8	0.07	95.3
PPG 1000 + DIMCARB + water	11	92.9	−2.73	−1.397	0.047	78.2	0.15
	12	94.8	−2.96	−0.429	0.003	79.4	0.09
	13	95.8	3.10	0.609	307.1	0.08	98.2
	14	96.7	3.05	0.493	358.9	0.09	96.6
	15	97.4	3.20	0.105	1237	0.14	98.8

a *Data taken from literature (Song et al., 2018b)*.

b *K values are expressed as a mean of triplicate readings with the estimated error of < 5%*.

However, an inverse trend of partition behavior of GFP was noted in some of the systems (sample number: 6 7, 11, and 12), which are reflected by the negative values of log *K*_GFP_ (see Table 1). The compositions of systems undergone the phase inversion were analyzed, and the results are summarized in Table 2. From the liquid-liquid equilibrium data of these systems, the top phase mainly consisted of DIMCARB, whereas PPG was predominantly concentrated in the bottom phase. Prior to the addition of feedstock to these systems, the concentration of PPG 700/1000 was in the range of 44–50% (w/w) and the concentration of DIMCARB was ≤ 3% (w/w). The phase inversion occurred upon the addition of feedstock to these systems. As shown in Table 2, the addition of feedstock reduced the concentrations of PPG 700/1000 in both phases. The phase inversion may be associated to the temperature-responsiveness of PPG; the lower critical solution temperature (LCST) of PPG 700 and PPG 1000 may increase with a decreasing concentration of polymer. At room temperature and a lower concentration of PPG 700/1000, the hydrophobic moieties along the polymer chains were suspected to experience desolvation, resulting in the polymer aggregates, and a denser polymer-rich phase. Therefore, the inversion of phases occurred in the system and rendered the DIMCARB-rich fraction as the top phase.

**Table 2:** Compositions of the selected PPG 700/1000 (1) + DIMCARB (2) + water (3) systems at 25°C and 101.32 kPa.

**Sample number**	**Total compositions**		**Without crude feedstock**				**With crude feedstock^b^**			
			**Top phase^a^**		**Bottom phase^a^**		**Top phase^c^**		**Bottom phase^c^**	
	100 *w*_1_	100 *w*_2_	100 *w*_1_	100 *w*_2_	100 *w*_1_	100 *w*_2_	100 *w*_1_	100 *w*_2_	100 *w*_1_	100 *w*_2_
**PPG 700** + **DIMCARB** + **water**										
6	46.0	3.0	94.2	0.58	1.40	5.35	1.08	5.87	87.8	0.68
7	44.0	2.5	92.7	0.59	2.70	4.14	2.32	4.48	84.0	0.74
**PPG 1000** + **DIMCARB** + **water**										
11	50.0	1.7	95.0	0.41	2.14	3.11	1.70	4.03	88.2	0.52
12	48.0	2.3	95.6	0.40	0.87	4.31	0.65	5.54	88.8	0.55

a *Data taken from literature (Song et al., 2018b)*.

b *The concentrations of PPG, DIMCARB, and water in both top and bottom phases of the investigated ATPSs were analyzed using the methods described in previous study (Song et al., 2018b)*.

c *Standard uncertainty of temperature = 1°C and pressure = 0.5 kPa. Expanded uncertainty: for PPG 700 + DIMCARB + water system, Uc are Uc(PPG 700) = Uc(DIMCARB) = 0.0021 (95% level of confidence); for PPG 1000 + DIMCARB + water system, Uc(PPG 1000) = Uc(DIMCARB) = 0.0023 (95% level of confidence)*.

Figure 3 shows that the volumes of the top phases of PPG 400 + DIMCARB + water + feedstock systems decreased with an increasing TLL. This indicated that the equilibrium of these PPG400-based systems was affected by the addition of feedstock. In contrast, the volume ratio of PPG 700/1000 + DIMCARB + water + feedstock systems remained mostly constant. Furthermore, the amount of noticeable protein precipitate at the interphase of PPG 400 + DIMCARB + water + feedstock systems increased as the TLL of the system increased. This may be attributed to a higher degree of salting-out of host cell proteins in the systems. The presence of protein debris was also found at the interphase of ATPSs composed of PPG 700/1000. Nonetheless, the precipitation of the host cell protein in these systems also served as a means for the removal of protein contaminant, thereby assisting in the purification of GFP by ATPS.

### Recovery of phase-forming components

Despite the promising potential of IL-based ATPS for the application in protein separation, these systems are yet to be widely adopted in the industrial operations due to the high cost of IL. In contrast to the conventional ILs, DIMCARB is relatively cheaper because of the use of CO_2_ as the raw material. Nevertheless, the cost of DIMCARB is still about 2 to 5 times higher than that of the conventional phase-forming components (e.g., inorganic salts, alcohol and carbohydrates). Therefore, the recycling of IL for the practical application of ATPS is highly desirable.

The recyclability of DIMCARB was evaluated using the optimized ATPS composed of 42% (w/w) PPG 1000 and 4.4% (w/w) DIMCARB. Firstly, the scale of ATPS was increased from 2 g to 100 g. The compositions and purification efficiencies of the scaled-up system are presented in Table 3. Regardless of the scale of ATPS, the composition of the system and the purification efficiencies remained nearly identical. The results affirmed that the performance of GFP purification was not compromised by the scalability of this IL-based ATPS. DIMCARB can be easily distilled and recovered via evaporation (Song et al., 2018b). In this study, the DIMCARB-rich bottom phase of the 100-g ATPS containing the partitioned GFP was subjected to rotary evaporation at 45°C and 85 mbar for 1 hr. During the process, DIMCARB dissociated into the gaseous dimethylamine and CO_2_. As the gasses passed through a condenser unit, the re-association occurred and the DIMCARB was reformed in the liquid state.

**Table 3:** Compositions and purification efficiencies of 2- and 100-g ATPSs comprising 42% (w/w) PPG 1000, 4.4% (w/w) DIMCARB, and 10% (w/w) feedstock at pH 8.

**Total system weight (g)**	**Compositions**				***S***	***Y*** **(%)**	
	**Top phase**		**Bottom phase**		
	**100 *w*_1_**	**100 *w*_2_**	**100 *w*_1_**	**100 *w*_2_**		**Top phase**	**Bottom phase**
2	97.6	0.37	0.40	7.82	1237	0.14	98.9
100	96.9	0.32	0.34	7.71	1229	0.13	98.3

**Figure 4:**
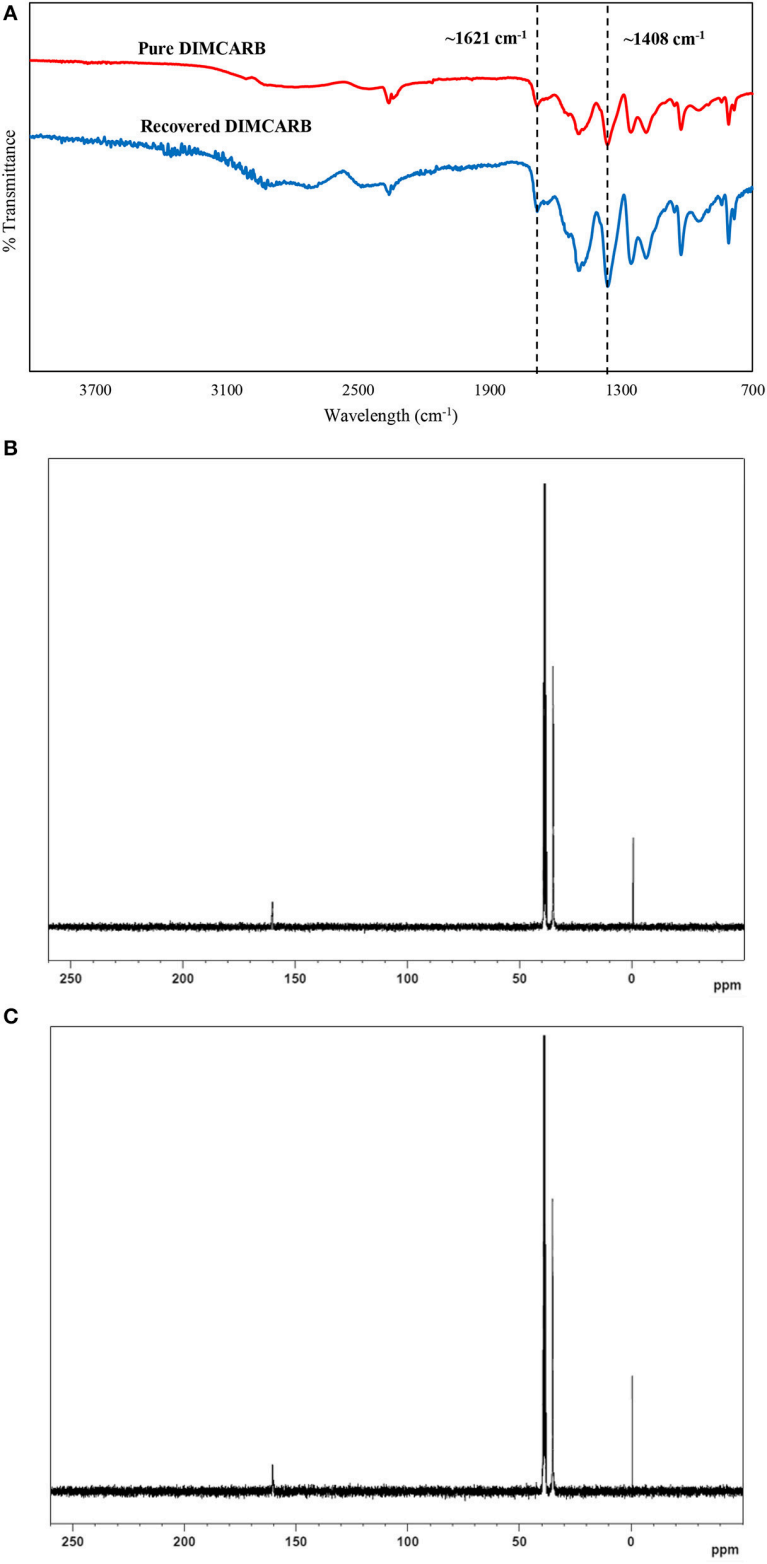
Characterization of DIMCARB. **(A)** FT-IR spectra of the pure DIMCARB and the DIMCARB recovered from distillation; **(B)** ^13^C NMR spectra of the pure DIMCARB; **(C)** ^13^C NMR spectra of the DIMCARB recovered from distillation.

**Figure 5:**
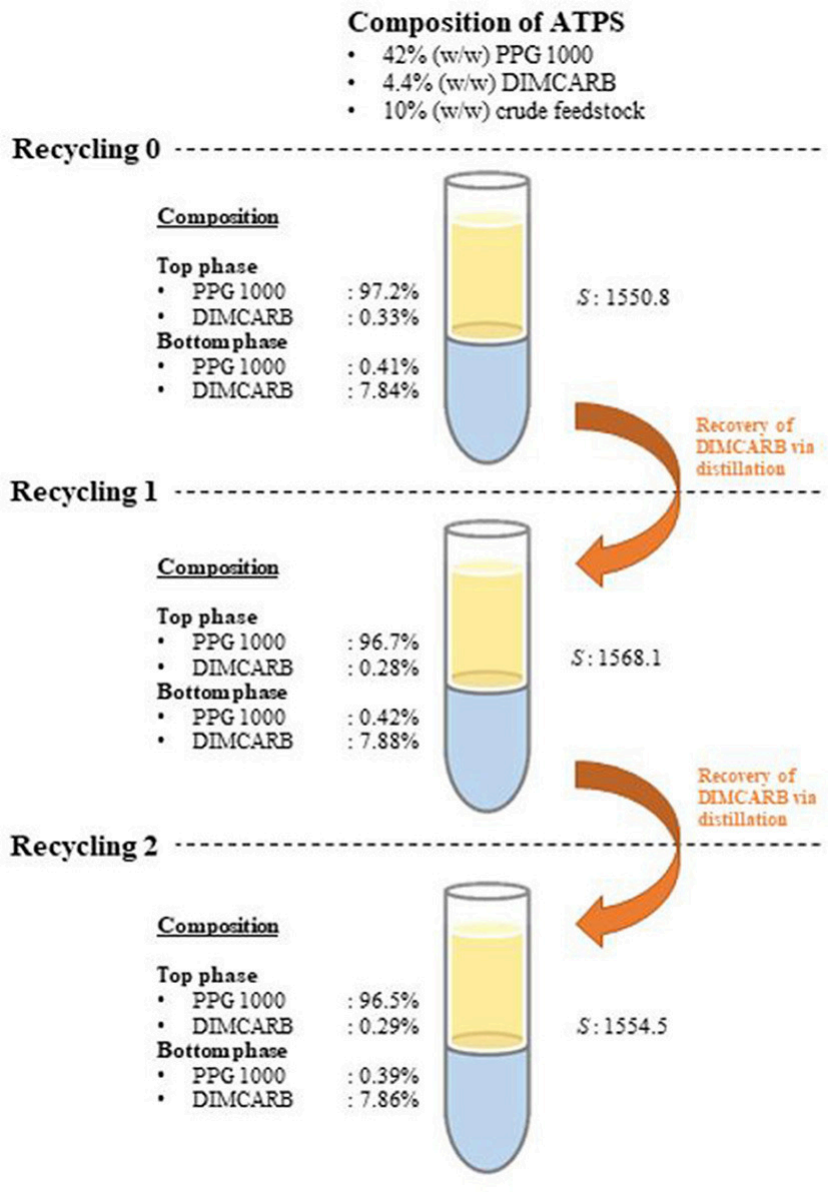
Schematic diagram of the recycling ATPSs for three successive rounds of GFP purification.

The DIMCARB recovered from the distillation was characterized by Fourier Transform-Infrared (FT-IR) and carbon-13 nuclear magnetic resonance (^13^C NMR) spectroscopies. The results of FT-IR and ^13^C NMR analyses are presented in Figure 4. In the FT-IR spectra, the symmetric carbamate (~1408 cm^−1^) and the carbamate C–O stretching (~1621 cm^−1^) peaks were noted in both pure and recycled DIMCARB samples (see Figure 4A). Similarly, the carbamate signal at ~162 ppm was observed in the ^13^C NMR spectra of pure and recovered DIMCARB samples (see Figures 4B,C). These analyses confirmed the successful recovery DIMCARB from the bottom phase of the ATPS. Moreover, the percentage relative concentration of GFP before and after the distillation of DIMCARB was found to be 99.4% (data not shown), indicating that the stability of GFP was well preserved during the distillation of DIMCARB. The DIMCARB recovered from the distillation was used to prepare the new batch of ATPS for use in GFP purification. As illustrated in Figure 5, this recycling step was performed for three successive rounds of GFP purification, which is denoted as Recycling 0, Recycling 1, and Recycling 2, respectively. Overall, the phase compositions of the recycling ATPSs were almost similar to that of the primary ATPS. Moreover, the partition behavior of GFP in the recycling ATPSs did not deviate significantly (as indicated by the *S*-values shown in Figure 5).

### SDS-PAGE analysis of purified GFP using PPG + DIMCARB + water systems

**Figure 6:**
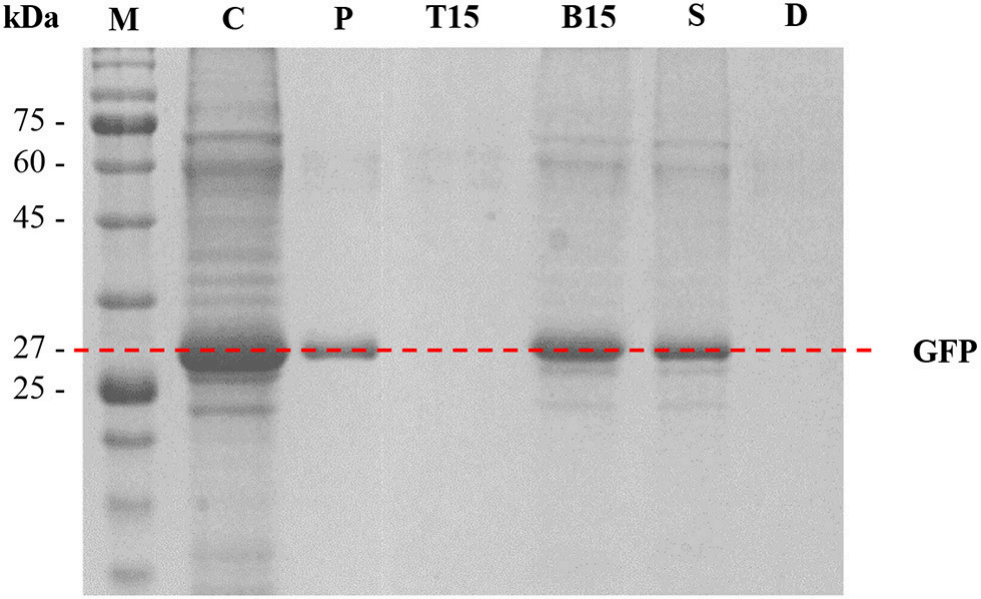
SDS-PAGE analysis. Lane M, protein marker; Lane C, crude feedstock; Lane P, GFP standard; Lanes T15 and B15, the top phase and bottom phase of the optimized ATPS (sample number 15, as stated in Table 1), respectively; Lane S, distilled bottom phase of 100-g ATPS; Lane D, distillate obtained from the recovery of DIMCARB.

SDS-PAGE analysis was performed to assess the purity of protein and the performance of protein separation by the ATPSs. The results are shown in Figure 6. In Lane P, GFP standard was identified as a single thick band at approximately 27 kDa. In Lane C (crude feedstock sample), multiple protein bands were detected along with a thick band of GFP at 27 kDa, indicating the presence of protein impurities in the harvested culture broth prior to the purification process. For the optimized ATPS made of 42% (w/w) PPG 1000 and 4.4% (w/w) DIMCARB (sample number 15), the top phase of the system (Lane T15) did not exhibit any protein band. This hinted that the protein impurities had been mostly precipitated and concentrated at the interphase of the system (see Figure 3). On the other hand, the bottom phase of the system (Lane B15) showed a single dark band at ~27 kDa (i.e., GFP) and some faint bands representing minor impurities. Similarly, the bottom phase from the scaled-up ATPS (Lane S) had the similar profile of protein bands as Lane B15. The Lane D representing distillate recovered from the rotary evaporation of DIMCARB-rich phase did not show any protein band.

## Conclusions

The PPG + DIMCARB + water systems were successfully applied for the purification of GFP from the clarified *E. coli* lysate. In general, GFP has a higher affinity toward the DIMCARB-rich phase. The optimal purification of GFP was attained with an ATPS composed of 42% (w/w) PPG 1000 and 4.4% (w/w) DIMCARB. The optimized ATPS was also successfully scaled up by 50 times. Moreover, DIMCARB was successfully recovered from the IL-rich phase and was reused for three successive rounds of GFP purification. Overall, PPG + DIMCARB + water system has demonstrated the satisfactory performance in the purification of protein from microbial lysate. The ease of recycling DIMCARB via distillation makes the ATPS even more sustainable and environmentally benign for application in protein purification.

## Author contributions

The experiments were designed by CS, PL, and CO. The experiments were carried out by ZT and SL. The manuscript was written by CS, PL, PS, and CO. All authors have read and approved the final manuscript.

### Conflict of interest statement

The authors declare that the research was conducted in the absence of any commercial or financial relationships that could be construed as a potential conflict of interest.
